# Narratives of resilience: Understanding Iranian breast cancer survivors through health belief model and stress-coping theory for enhanced interventions

**DOI:** 10.1186/s12905-024-03383-7

**Published:** 2024-10-08

**Authors:** Mohaddese Mehrabizadeh, Zeinab Zaremohzzabieh, Mansoureh Zarean, Seyedali Ahrari, Ali-Reza Ahmadi

**Affiliations:** 1 Women and Family Studies Research Center, University of Religions and Denominations, Qom, Iran; 2 Women Research Center, Alzahra University, Tehran, Iran

**Keywords:** Breast cancer, Survivors, Adaptation, Coping strategies, Health Belief Model, Lazarus’ stress-coping theory

## Abstract

Breast cancer poses a significant global health challenge, with Iran experiencing particularly high incidence and mortality rates. Understanding the adaptation process of Iranian breast cancer survivors’ post-treatment is crucial. This study explores the health perceptions, barriers, and coping mechanisms of Iranian survivors by integrating Stress-Coping Theory (SCT) and the Health Belief Model (HBM). Semi-structured interviews were conducted with 17 survivors, and a grounded theory approach guided the deductive content analysis of the data. The findings reveal key themes, including perceived susceptibility, benefits, barriers to care, cues to action, self-efficacy, and appraisal of action. Perceived susceptibility highlights diagnostic challenges stemming from practitioner errors and symptom misconceptions. Perceived benefits underscore the importance of early detection and support from healthcare providers and families. Barriers include cultural and financial obstacles, while cues to action reflect the influence of media, family, and personal experiences on healthcare-seeking behavior. The study also examines coping strategies, such as problem-focused and emotion-focused approaches, along with family support and external stressors. To address these barriers and enhance support systems, the study suggests specific strategies for healthcare providers, including targeted training to improve diagnostic accuracy and patient communication. Culturally sensitive awareness campaigns can correct symptom misconceptions, while financial counseling can mitigate economic barriers. Establishing community-based support groups and involving family members in care plans can enhance emotional and psychological support. These strategies aim to overcome the identified barriers and improve support systems for Iranian breast cancer survivors, ultimately fostering better recovery outcomes.

**Supplementary Information:**

The online version contains supplementary material available at 10.1186/s12905-024-03383-7.

## Introduction

Breast cancer remains one of the most prevalent cancers among women worldwide, and Iran is no exception [1–4]. Among Iranian women, the 5-year prevalence of breast cancer is 37.7%, with age-standardized incidence and mortality rates estimated at 33.21 and 14.2 per 100,000 population, respectively [5]. Despite advances in medical treatments, survivors often face substantial psychological and social challenges post-treatment, significantly affecting their quality of life [6–8]. Developing effective interventions to support these survivors requires a comprehensive understanding of their unique experiences and coping mechanisms. To investigate the distinct experiences and coping strategies of breast cancer supervisors, several theoretical frameworks have been employed [9–11].

Prominent among these are Lazarus’ theory of appraisal, stress, and coping [12], as well as the Health Belief Model [13]. These models provide valuable insights into how individuals perceive and manage the stress associated with their roles, offering a comprehensive understanding of their unique challenges and adaptive mechanisms. According to Lazarus’ theory, coping is constantly changing cognitive and behavioral efforts to manage specific external and/or internal demands perceived as stressors [14]. Coping strategies are classified as “problem-focused coping,” which includes behaviors aimed at solving the problem, such as active coping, planning, seeking social (instrumental) support, and suppression of emotions [15]. Additionally, “emotion-focused coping” involves behaviors directed at changing emotional reactions to problems or situations, such as seeking social (emotional) support, turning to religious support, and denial [16].

Cultural beliefs and values are increasingly recognized as important determinants of psychological and behavioral outcomes following cancer diagnosis and treatment [17, 18]. Survivors’ behaviors are influenced by their perceptions of the disease, and studies show that their beliefs about cancer causation lead them to change behaviors to prevent recurrence [19, 20]. The HBM provides a framework for understanding how individuals perceive health risks and the factors influencing their engagement in health-promoting behaviors. Meanwhile, Stress-coping Theory (SCT) elucidates how individuals manage and adapt to stressful life events, which is critical for designing supportive interventions for cancer survivors [21]. By integrating these theoretical perspectives, this study provides a comprehensive framework that not only captures individuals’ health perceptions and behaviors but also encompasses the critical emotional and stress-related dimensions of coping with breast cancer.

Until now, several qualitative studies have investigated the challenges faced by women with breast cancer in Iran [19–25]. These studies have varied in scope, timeframe, and focus, examining aspects such as positive life changes, spiritual dimensions, feelings of pity, support systems, and issues related to childbearing age. However, there remains a gap in research regarding the combined application of the HBM and SCT in understanding the adaptation process of Iranian breast cancer survivors. In Iran, where cultural norms and healthcare practices differ significantly from Western contexts, understanding how breast cancer survivors perceive their health and cope with stress is crucial for developing effective and culturally sensitive interventions. Unlike the Western emphasis on individualism and personal autonomy in health decisions, Iranian society often prioritizes collective responsibility and familial involvement in healthcare [22]. This collectivist orientation shapes unique coping strategies, where emotional support from family members plays a central role, contrasting with the more self-reliant approaches commonly observed in Western societies [23]. Additionally, the perception and management of mental health in Iran are heavily influenced by cultural stigma, which frequently discourages open discussions about psychological distress. As a result, breast cancer survivors may express their stress through somatic complaints rather than seeking direct mental health support [24]. This somatization of psychological distress highlights the critical need for culturally adapted interventions that address both the physical and emotional well-being of survivors.

This study explores the applicability of the HBM and SCT in Iranian breast cancer survivors, aiming to advance intervention strategies to improve health outcomes and behaviors. This qualitative approach offers rich, detailed insights into the psychological and emotional landscapes of these survivors, facilitating the development of culturally sensitive and contextually relevant interventions. By grounding this study in well-established theoretical frameworks, we aim to contribute to the body of knowledge on survivorship care and provide practical recommendations for healthcare providers working with breast cancer survivors in Iran.

## Literature review

### Health belief model

HBM is a well-established theoretical framework designed to elucidate why individuals engage in preventive health behaviors. Developed by Becker [13], the model is grounded on six primary constructs: (1) perceived susceptibility to the health condition, (2) perceived severity of the health condition, (3) perceived benefits of taking the recommended action, (4) perceived barriers to taking the recommended action, (5) cues to action to engage in the preventive health behavior, and (6) self-efficacy to engage in the preventive health behavior.

The HBM has become one of the most widely utilized frameworks for understanding health intervention uptake and behavior, proving invaluable in the planning and design of various health interventions [25, 26]. For instance, Darvishpour et al. [27] used the HBM to investigate predictors of breast cancer screening behaviors. Similarly, Ramezankhani et al. [28] examined the reasons symptomatic women delay seeking early screening for breast cancer using HBM. Additionally, Wondmu et al. [29] assessed the impact of HBM-based breast cancer education on knowledge, health beliefs, and breast self-examination practices among female students in Ethiopia.

In the context of breast cancer survivors, the HBM offers a critical perspective for comprehending how their health behaviors and coping strategies are influenced by their perceptions of susceptibility, severity, benefits, barriers, cues to action, and self-efficacy [30–32]. Although the HBM has been extensively used to develop and implement prevention programs, it has not been used comprehensively to identify themes and index meaning units from primary qualitative data to understand breast cancer patients’ attitudes and beliefs on engaging in health prevention. Specifically, the application of HBM constructs to explore breast cancer patients’ perceptions about engagement in health prevention behaviors presents a novel approach. By assessing their perceived susceptibility to breast cancer recurrence, perceived severity of the consequences, perceived benefits of taking action, perceived barriers to action, and recommended cues to action, we can gain a deeper understanding of the factors influencing their adherence to health prevention behaviors.

### Stress-coping theory

The theoretical framework for this study consists of perspectives on coping previously applied in breast cancer research as reflected in Lazarus’ [12] theory of appraisal, stress, and coping. According to Lazarus and Folkman [33], coping involves persistently changing cognitive and behavioral efforts to manage specific external and/or internal demands that are seen as taxing or exceeding one’s resources. The stress associated with a situation depends on an individual’s appraisal of the situation and the behaviors directed at managing the stress. Lazarus [12] identified two major types of coping strategies: (i) Problem-focused coping lifestyle, which aims at solving the problem or situations, and (ii) Emotion-focused/ avoidant coping lifestyle, aimed at regulating the emotions related to stress. Problem-focused coping includes obtaining relevant information about what to do, whereas emotion-focused includes avoiding thinking about the threat or reappraising it without changing the realities of stressful situations. The two coping strategies can both facilitate and impede each other throughout a stressful situation.

Cancer patients, including breast cancer survivors, tend to adjust to the burden of disease through a wide range of coping strategies [34–36]. These strategies can significantly vary in their effectiveness and impact on the individual’s well-being [37]. Using coping strategies can influence psychological adjustment, quality of life, and overall survivorship experience. By integrating the SCT with the HBM, this study aims to develop a nuanced understanding of the coping mechanisms employed by breast cancer survivors. This dual-theoretical approach allows for a comprehensive analysis of how health beliefs influence coping strategies and, subsequently, health behaviors. The insights gained from this approach can inform the design of targeted interventions that support breast cancer survivors in managing stress and promoting healthier coping mechanisms, ultimately improving their quality of life and long-term outcomes.

## Methods

### Research design

**Fig. 1:**
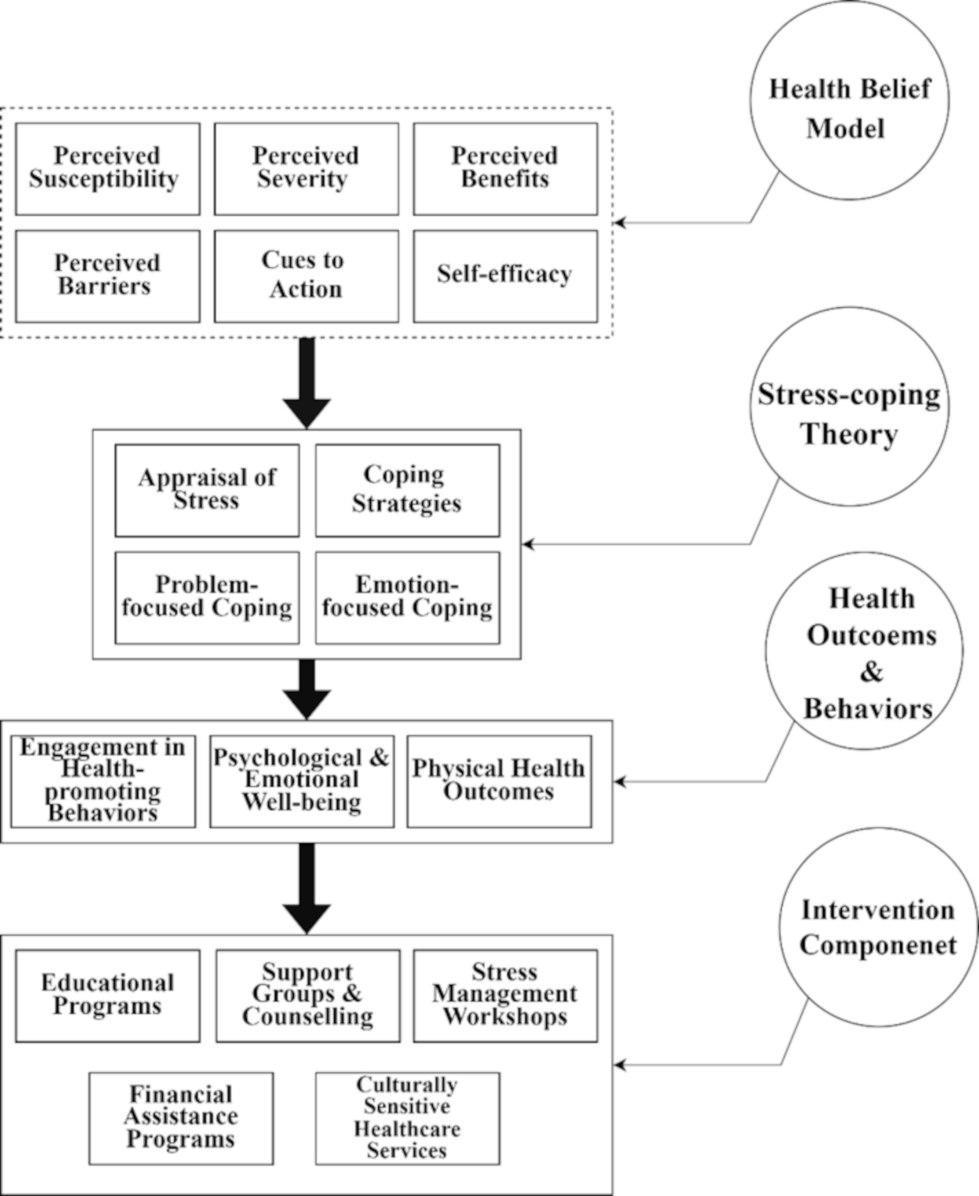
Theoretical framework of this study

This study employed an informed grounded theory approach to explore breast cancer survivors’ perceptions of health risks, health-promoting behaviors, and strategies to enhance engagement in such behaviors, utilizing constructs from the HBM and SCT. Informed Grounded Theory [IGT; 38] allows for the incorporation of existing literature and theoretical frameworks to guide qualitative research. The HBM and SCT were integral in understanding the psychological and emotional experiences of the survivors, aiding in the creation of culturally sensitive and contextually relevant interventions (Fig. 1).

These theories guided the development of the semi-structured interview guide (Table 1), drawing from established research [39, 40]. Questions 1 and 2 were designed to assess participants’ perceptions of their risk and the seriousness of breast cancer, corresponding to HBM’s constructs of perceived susceptibility and severity. Questions 3 and 4 addressed perceived benefits of medical interventions and obstacles to seeking treatment, reflecting HBM’s focus on perceived benefits and barriers. Question 5 explored triggers that motivate participants to seek medical help, aligning with HBM’s cues to action. Question 6 assessed participants’ confidence in managing and overcoming breast cancer, reflecting SCT’s self-efficacy construct. Questions 7 and 8 examined stress related to diagnosis and treatment, and techniques used to handle stress, related to SCT’s outcome expectations and coping strategies.

To refine the interview guide, three preliminary one-on-one interviews were conducted. The feedback from these interviews was used to adjust the guide, although the initial interviews are not included in the final study analysis. Before participating in the interview, all participants completed a questionnaire detailing their demographics and medical history. This preparatory step ensured a comprehensive understanding of the participants’ backgrounds, facilitating more focused and relevant discussions during the interview.

**Table 1:** Semi-structured interview guide

No.	Queries
1	How do you perceive their risk?
2	How serious do you believe the disease to be?
3	What positive outcomes do you associate with medical interventions?
4	What obstacles do you perceive in seeking treatment?
5	What triggers motivate you to seek medical help?
6	How confident are you in managing and overcoming breast cancer?
7	How do you assess the stress related to their diagnosis and treatment?
8	What techniques do you use to handle stress?

### Sampling

This research focused on women in Isfahan, Iran, who had undergone treatment for breast cancer. Using purposive sampling, participants were chosen based on strict inclusion criteria: they had to be newly diagnosed, currently undergoing or having completed treatment, informed of being cancer-free, married, and over 18 years old. Individuals with a history of major mental health issues were excluded from the study. Recruitment was carried out through a homecare nurse and a medical center affiliated with Isfahan University of Medical Sciences. In-person interviews were scheduled at the participant’s convenience, and data collection continued until data saturation was reached.

### Data collection

Data collection involved semi-structured interviews with 17 patients. The decision to include 17 participants was based on achieving data saturation, a key consideration in qualitative research. Data saturation refers to the point at which no new information or themes are emerging from the data, and further data collection no longer contributes to the understanding of the research topic [41]. In our study, data saturation was reached with 17 interviews.

The interviews, conducted by the first author, took place between May and October 2023. Each interview lasted approximately 60 min and was digitally recorded. The questions, derived from a literature review [42–44], were validated by an expert panel of three lecturers from two famous universities in Tehran, Iran. These experts selected based on their experience, qualifications, and expertise in qualitative research (1st & 2nd experts) and the current research topic (3rd expert). Their insights and perspectives provided valuable information, context, and depth to the present study methods and findings.

The interviews’ verbatim transcription occurred within 48 h of each session. Participants received a gift card. This study was approved by the university’s Ethics Committee for Research Involving Human Subjects (ref no: IR. REC.1398.167). All research was conducted by the Declaration of Helsinki, ensuring that participants’ rights and well-being were prioritized and protected throughout the study.

The identities of the respondents were kept confidential on the information form. By the Declaration of Helsinki, all research participants’ rights and well-being will be prioritized and protected throughout the study. In the beginning, the purpose and research method were explained to the interviewees. Each participant was informed about the rights and principles of protection of human subjects. They were assured that their information would be kept confidential, and measures were taken in this regard, including the use of aliases for recording information.

### Data analysis

Demographic data were processed using Excel for descriptive statistics. Interviews were transcribed and edited for accuracy, followed by deductive content analysis using the HBM and SCT as frameworks. Key steps included identifying core constructs for initial coding, categorizing meaning units, and achieving consensus on themes. Trustworthiness was ensured through member checking and investigator triangulation, involving two expert researchers who independently coded the data and reached an agreement through discussion. Data saturation was confirmed after four focus groups.

## Results

### Sample description

The demographic data of the participants is summarized in Table 2. The participants’ ages ranged from 26 to 48 years, with varying levels of education from middle school to master’s degrees. Most participants were married or had been married, with the majority having children. Their financial status varied from poor to good, and their support systems included spouses, families, or in some cases, no support at all. Several participants had a family history of cancer, while others did not. The table provides a detailed overview of their demographic characteristics.

**Table 2:** Participant demographics

Participant	Age	Education	Demographics
1	32	Bachelor	Has 1 child. Her husband has accompanied her. He is a doctor. Their financial status is good. No family history.
2	43	Diploma	Married with 3 children. Tenant. The husband is a worker. No family history of cancer. Lacks support.
3	43	Bachelor in psychology	A single mother with 2 children and, a family history of cancer (father). Her father passed away from cancer, causing concern about mortality. Her brother provided support.
4	47	Master in Persian language	A single mother with one married child and a grandchild. Divorced before illness, good financial status. Remarried late in treatment.
5	43	Bachelor	Discovered during early marriage while she was pregnant. She works in an office. She is a tenant. Her husband has accompanied her, and the family has provided support.
6	42	Bachelor	Has 2 children. Financial status is good. The husband supported her but not much initially. Family history of illness. Housewife.
7	45	Bachelor	Has 1 child. Discovered during play with the child. Teacher. Financial status is moderate. No family history of illness.
8	48	Middle school	Has 3 children. Housewife. Tenant. Financial status is poor. Husband and family supported her.
9	48	High school	Has 2 children. Employed. Financial status is moderate. Owns an independent home.
10	37	Bachelor	Has 1 child. Housewife. The husband is an employee. The husband’s family did not accompany her. Tenant. No family history of illness.
11	27	High school	Discovered while single. Family supported. Engaged in low-income home jobs. Financial status is moderate. Concern about marriage. Removed one breast.
12	42	Diploma	Has 1 child. Housewife. The husband is an employee. Has own home. No family history of illness.
13	38	Bachelor	Has 1 child. Employee. Illness during COVID-19. Due to disease restrictions, no one accompanied her. Financial status is good. The husband supported her emotionally.
14	43	High School	Single. No support. Unemployed. Financial status is poor.
15	29	Associate Degree	Engaged during illness. Aunt had a previous illness and, a family history. Husband supported. Did not inform families during treatment. No children yet.
16	27	Bachelor	Aunt was ill. Discovered illness during the engagement. Financial status is moderate. Husband supported. Has income. Her husband is a teacher.
17	35	Diploma	Single. Has two children in the family. Financial status is good. Family supported. Unemployed.

### Emerged themes

Table 3 illustrates the themes and subthemes that emerged from the study, highlighting the diverse factors influencing breast cancer detection and treatment among participants. The table organizes these insights into major themes such as perceived susceptibility, perceived benefits, perceived barriers to breast cancer care, cues to action, self-efficacy, appraisal of stress, and coping strategies. Each theme is further broken down into subthemes that capture the nuanced experiences and perceptions of the participants.

**Table 3:** Emerged themes and subthemes in breast cancer detection and treatment

Theme	Subtheme
Perceived Susceptibility	Failure and Incorrect Diagnosis by GPs
	Discovering the Disease Despite General Health
	Assumptions and Beliefs About Disease Affliction
Perceived Benefits	Importance of Early Detection and Treatment
	Positive Outcomes from Medical Interventions
	Support and Encouragement from Family and HCPs
Perceived Barriers to Breast Cancer Care	Shame and Sensitivity in Consulting Male Doctors
	Financial Stress and Treatment Costs
	Fear of Body Image Changes
Cues to Action	Influences of Media and Information Sources
	Encouragement from Family and Religious Beliefs
	Personal Triggers and Experiences
Self-Efficacy	Cognitive Adaptation and Acceptance
	Overcoming Challenges and Resilience
	Support Systems Enhancing Coping Mechanisms
Appraisal of Stress	Importance of Timely Diagnosis
	Need for Improved Diagnostic Measures
Coping Strategies	Problem-Focused Coping
	Emotion-Focused Coping
	Emotional Venting
	Support from Family Members
	External Stressors

Note. GPs = General Practitioners

### Perceived susceptibility

The theme of perceived susceptibility in breast cancer diagnosis highlights significant challenges and misconceptions affecting early detection and treatment. This theme is divided into three subthemes:

1. **Failure and Incorrect Diagnosis by General Practitioners** A major concern is the “failure and incorrect diagnosis by general practitioners.” For instance, one participant described her experience: “*After my arm went numb*,* I visited a general practitioner who advised me to get an ultrasound. The result showed no serious issue*,* and he attributed my symptoms to vitamin deficiency. I felt relieved*,* but this misdiagnosis delayed the accurate detection of my condition*.” This account illustrates a frequent issue where symptoms are attributed to less severe conditions, causing delays in identifying serious illnesses like breast cancer. Such delays can lead to disease progression, highlighting the need for primary care physicians to improve their diagnostic accuracy and thoroughness.
2. **Discovering the Disease Despite General Health** Another participant discussed “discovering the disease despite general health,” revealing the unexpected nature of breast cancer detection. She noted: “*During a routine breast self-exam*,* I felt a firm lump that I hadn’t noticed before. Despite feeling generally healthy*,* I decided to see a physician who recommended surgery to test the lump*,* though they didn’t initially suspect cancer*.” This case underscores the importance of regular self-examinations and the potential for breast cancer to develop unnoticed, emphasizing the need for timely medical evaluations even when one feels well.
3. **Assumptions and Beliefs About Disease Affliction** The subtheme “assumptions and beliefs about disease affliction” highlights common misconceptions about breast cancer symptoms and risk factors. One participant shared: “*When I experienced severe chest pain*,* my physician questioned why I waited so long to seek help*,* pointing out that family history or pain severity were not always indicative of breast cancer*.” These narrative reveals misconceptions that pain and family history should dictate disease risk, stressing the need for broader education on the varied symptoms of breast cancer. Recognizing and addressing symptoms promptly, irrespective of pain or family history, is crucial for early diagnosis.

### Perceived benefits

This theme examines the perceived advantages of early detection and treatment of breast cancer, successful medical interventions, and support from families and healthcare providers. The subthemes include:

1. **Importance of Early Detection and Treatment.** The significance of early detection and timely medical consultation is highlighted by a participant’s experience. She discovered a lump during a routine self-exam, initially disregarded it, but later sought medical advice. Her physician recommended surgery and testing, demonstrating the critical role of self-exams and prompt medical intervention in managing breast cancer effectively. This case underscores how early detection can lead to better health outcomes.
2. **Positive Outcomes from Medical Interventions.** Effective medical interventions can foster psychological resilience and empowerment in breast cancer patients. One participant shared her determination to “fight this disease” following her diagnosis, influenced by past hardships. Her proactive approach and positive attitude illustrate how medical support can enhance patient resilience and adherence to treatment plans, highlighting the importance of maintaining a positive mindset in managing the disease.
3. **Support and Encouragement from Family and Healthcare Providers.** Emotional support from family and healthcare providers is crucial in the recovery process. One participant described how her husband’s reassurance and simple gestures, such as bringing her a glass of water and expressing his relief, significantly eased her emotional burden. This support was perceived as a key component of her healing journey, emphasizing the vital role of family encouragement in enhancing psychological well-being and contributing to overall recovery.

### Perceived barriers to breast cancer care

This theme examines the obstacles women face in accessing timely and effective breast cancer care, as illustrated by their personal experiences. The subthemes are:

1. **Shame and Sensitivity in Consulting Male Doctors.** Cultural and religious factors can significantly impact health-seeking behaviors. One participant described her reluctance to consult a male physician due to cultural and religious reasons, which she felt exacerbated her condition. This underscores the need for culturally sensitive healthcare approaches to address societal norms that may hinder timely medical care.
2. **Financial Stress and Treatment Costs.** Financial barriers are a major impediment to accessing breast cancer care. One participant shared her struggle with the economic burden of treatment, compounded by criticism from her spouse’s family about the perceived futility of spending money on her illness. This highlights the necessity for better financial support mechanisms and efforts to change attitudes towards funding cancer treatment to improve patient access and outcomes.
3. **Fear of Body Image Changes.** The emotional impact of body image changes due to cancer treatment is a significant barrier. One participant recounted her distress upon seeing the physical changes after surgery, which initially led her to seek privacy and cry. Although she has learned to cope with these changes, this experience underscores the need for support systems that address both the physical and emotional challenges of treatment.

### Cues to action

The theme of “cues to action” encompasses various factors motivating individuals to seek medical attention and engage in preventive health measures. These factors include influences from media and information sources, encouragement from family and religious beliefs, and personal triggers and experiences.

1. **Influences of Media and Information Sources.** The role of media and information sources significantly impacts health behaviors. Accurate information can lead to early diagnosis and treatment, whereas misinformation or lack of information can delay medical help. Participants noted that media campaigns and informative articles played a crucial role in their health-seeking behaviors. For example, one participant stated, “*The news segment on breast cancer awareness prompted me to schedule my first mammogram the next day*.” Similarly, social media campaigns and health articles were mentioned as key factors in encouraging regular check-ups.
2. **Encouragement from Family and Religious Beliefs.** Family support and religious beliefs often provide the emotional and psychological strength necessary to seek medical evaluations and treatments. Participants described how family members’ encouragement or religious teachings influenced their health decisions. One participant shared, “*My sister’s insistence on screening was a turning point for me.”* Another participant highlighted their faith, saying, *“My pastor’s health talk inspired me to prioritize my health*.“.
3. **Personal Triggers and Experiences.** Personal experiences and physical symptoms frequently act as significant triggers for seeking medical advice. These triggers include noticeable body changes or discomfort that prompt individuals to take action. Participants recounted how personal health scares or experiences with loved ones motivated them. For instance, one participant reflected, “*After a close friend’s death from breast cancer*,* I couldn’t ignore the lump in my breast*.” Another noted, “*The discomfort I felt in my breast became a pressing concern following my aunt’s diagnosis*.”

### Self-efficacy

Understanding self-efficacy is critical in comprehending how individuals respond to a breast cancer diagnosis and treatment. We have organized the subthemes related to self-efficacy as follows:

1. **Cognitive Adaptation and Acceptance.** Participants displayed a range of cognitive responses to their diagnosis, from initial denial to eventual acceptance. For instance, some participants initially refused to accept their illness, interpreting it as a mere illusion. One participant shared, “*When I found out…I didn’t want to accept being ill at all… I wished that the doctor and his words were just a dream*.” Over time, many participants adapted to their new reality, with one noting, “*I accept it… Every time I hear a new article about my illness…I accept it*.“.
2. **Overcoming Challenges and Resilience.** Participants demonstrated notable resilience and determination in overcoming the challenges posed by breast cancer. This resilience was often highlighted through their courageous responses to difficult situations. One participant expressed their resolve by stating, “*When the physician examined me and diagnosed the disease… I want to fight this disease because I’ve endured something worse than the disease in life*.“.
3. **Support Systems Enhancing Coping Mechanisms** Support from family members was crucial in enhancing participants’ coping mechanisms and self-efficacy. Emotional and affectionate support provided reassurance and helped patients manage the emotional challenges of their diagnosis and treatment. One participant reflected on this support, saying, “*At night when my husband came back… It was a moment that eased my heart*.”

### Appraisal of stress

We have organized the subthemes related to the appraisal of stress as follows:

1. **Importance of Timely Diagnosis** The appraisal of stress highlights the critical need for early diagnosis to mitigate the impact of breast cancer. Delays in detection can exacerbate the severity of the disease and its consequences for women’s health. As one participant noted, “*Delayed diagnosis is a major factor in the severity of breast cancer and its impact on women’s health*.“.
2. **Need for Improved Diagnostic Measures** Participants emphasized the need for enhanced diagnostic measures to address the issues contributing to late detection. One participant observed, “*The failure to diagnose breast cancer early significantly contributes to its severity and impact*.”

### Coping strategies

Among the coping strategies observed in individuals facing breast cancer, both problem-focused and emotion-focused approaches are employed to manage the illness’s challenges.

1. **Problem-Focused Coping.** Problem-focused coping includes proactive steps like seeking medical care and undergoing diagnostic tests. Many participants reported that their illness was discovered by chance due to a lack of previous screenings. As one participant noted, “*Several people mentioned that they had not had any prior tests*,* and their disease was found unexpectedly*.“.
2. **Emotion-Focused Coping.** Emotion-focused coping often involves minimizing the severity of symptoms and viewing the disease as temporary, even when experiencing significant discomfort. For example, one participant shared, “*Some individuals*,* despite feeling pain*,* tend to consider the illness as fleeting*,* struggling to accept the reality of their condition*.“.

This resistance to fully acknowledging the illness can complicate the coping process. As another participant described, “*I found it hard to accept being ill and couldn’t grasp what this illness meant or its consequences*.”

3. **Emotional Venting.** Emotional venting is used to manage the stress related to social acceptance and appearance changes. Participants expressed concerns about being judged by others due to their altered appearance. One participant said, “*I was very anxious about how my appearance might affect my relationships and social interactions*.“.
  Despite the hardships of breast cancer, individuals showed resilience and a strong will to fight the disease. One participant emphasized their determination, stating, “*When diagnosed*,* I resolved to fight the disease*,* drawing strength from overcoming previous life challenges*.”

4. **Support from Family Members.** Family support is a crucial factor in coping with breast cancer, providing emotional comfort and reassurance. However, some participants experienced neglect or a lack of support from family members, highlighting the need for better understanding and assistance. One participant reflected, “*Support from my family provided significant comfort*,* making me feel that I had made progress in my healing journey*.“.
5. **External Stressors.** External factors, such as maternal responsibilities, economic pressures, and living conditions, add to the stress of dealing with breast cancer. For instance, one participant expressed concerns about balancing family duties, saying, “*I want to be there for my son starting first grade*,* which adds to my stress*.” Another noted economic worries, stating, “*I constantly remind my husband about upcoming installment payments*,* which is an additional stressor*.”

## Discussion

This qualitative inquiry into the experiences of Iranian breast cancer survivors’ sheds light on key aspects of the HBM and SCT, offering valuable insights into their applicability in understanding health outcomes and behaviors within this demographic. Our study makes a unique contribution by specifically focusing on the experiences of Iranian breast cancer survivors, an area that has been underexplored in existing literature.

Our findings extend beyond the scope of previous research, such as Hassankhani et al. [45], which primarily emphasized the role of spiritual beliefs and family support in pain management. By integrating HBM and SCT, our study offers a broader perspective, exploring the determinants of health behaviors and highlighting the coping mechanisms employed by survivors as they navigate the challenges of breast cancer diagnosis and treatment. This integration of theoretical frameworks provides a comprehensive understanding of the complex interplay between cognitive, emotional, and social factors influencing health behavior among this population.

In contrast to Hassankhani et al.‘s [45] focus on spiritual and familial aspects, another study [46] on cancer pain self-management proposed a holistic model incorporating physical, psychosocial, cultural, and spiritual dimensions. Our research builds upon these findings by examining how resilience interacts with spiritual beliefs and family support in managing cancer-related distress. This exploration is crucial for developing culturally sensitive interventions tailored to the specific needs of Iranian breast cancer survivors.

The significance of this study lies in its potential to inform the development of interventions that are not only culturally sensitive but also holistically designed to meet the unique needs and preferences of Iranian women. Such interventions are critical for enhancing the well-being and quality of life of breast cancer survivors in Iran. The narratives from our participants underscore the importance of perceived susceptibility in breast cancer diagnosis, revealing challenges such as misdiagnoses by general practitioners, the discovery of the disease despite feeling generally healthy, and widespread misconceptions about the risk of affliction. These findings align with the HBM, which posits that individuals are more likely to engage in health-promoting behaviors if they perceive themselves as susceptible to a health threat and believe that preventive actions can mitigate this risk.

Additionally, the perceived benefits of early detection and treatment, positive outcomes from medical interventions, and the support from families and healthcare providers resonate with the HBM’s emphasis on perceived benefits as a key determinant of health behavior. Participants highlighted the importance of early detection through self-exams and the psychological resilience fostered by effective medical interventions and emotional support. This affirmation of the model’s relevance underscores the importance of understanding the health-seeking behaviors of breast cancer survivors.

The identified barriers to breast cancer care, including shame in consulting male doctors, financial stress, and fear of body image changes, underscore the necessity of addressing psychosocial and cultural factors that influence health behaviors. These barriers align with the HBM’s constructs of perceived barriers, which can hinder individuals from engaging in health-promoting behaviors despite recognizing the threat of breast cancer. Cultural factors such as shame, shyness, and stigmatization drive both male and female caregivers to prefer female physicians, potentially delaying treatment. Cultural myths regarding female beauty and body image also create reluctance among patients and caregivers to seek medical advice, especially concerning surgery and breast removal, which adds stress to both patients and caregivers who must manage their stress while providing emotional support [47, 48].

### Conflicting

Results have been observed in various studies concerning perceived susceptibility and its impact on breast cancer screening behaviors. For instance, Petro-Nustas [49] found that a higher level of perceived susceptibility was associated with increased breast cancer screening performance. Conversely, Foxall et al. [50] reported no relationship between breast cancer screening practices and perceived susceptibility or benefits among African-American and Caucasian nurses. Additionally, Gozum and Aydin [51] identified perceived benefits as a significant predictor of breast self-examination performance among Turkish women. These discrepancies highlight the need for further research to elucidate the diverse impacts of perceived susceptibility and benefits on breast cancer screening behaviors across different populations and cultural contexts.

Furthermore, the issue of “Failure and Incorrect Diagnosis by General Practitioners” is a frequently reported problem in Iran, as supported by the literature. Ghahramani et al. [52] identified one of the main barriers to women’s cancer screening in Iran as the failure of physicians to recommend screening. This finding aligns with our results and highlights broader systemic challenges within the Iranian healthcare system, particularly concerning the role of general practitioners.

Furthermore, many studies have revealed that the caregiving experience is complex and deeply embedded within cultural belief systems and social responses. Factors such as lower socioeconomic status, cultural norms of purdah (veil), shyness, stigmatization, and gendered ideologies significantly influence the experiences of both male and female caregivers [53]. These findings underscore the importance of considering cultural and socioeconomic factors when addressing breast cancer care and support, as they profoundly shape caregiving dynamics and health-seeking behaviors.

Cues to action, such as influences from media and information sources, encouragement from family, religious beliefs, and personal experiences, play a significant role in motivating individuals to seek medical attention and adopt preventive health measures. This is consistent with previous studies which suggest that strong religious beliefs are often used as a mechanism to alleviate tension, particularly in cases of critical illnesses like cancer [54, 55]. Religious beliefs can play a crucial role in shaping a patient’s approach to their illness, including their attitudes toward medical treatments, adherence to prescribed regimens, and engagement with healthcare providers. For instance, patients with strong religious convictions may perceive their illness as a test of faith or divine will, which could influence their coping strategies and openness to certain medical interventions. Understanding these dynamics could inform the development of culturally sensitive interventions that are more effective because they resonate with the patients’ spiritual and cultural context. These findings also support the HBM’s notion that cues to action prompt individuals to take preventive actions in response to perceived threats to their health.

Themes of self-efficacy and coping strategies shed light on breast cancer survivors’ cognitive adaptation, resilience, and use of problem-focused and emotion-focused coping mechanisms. Participants demonstrated various cognitive patterns upon diagnosis, ranging from denial to acceptance, and exhibited resilience in overcoming the challenges posed by breast cancer. These findings align with SCT, which emphasizes individuals’ appraisal of stress and their subsequent coping efforts to manage the demands of stressful situations.

Moreover, emotional venting emerged as a coping mechanism for individuals navigating the emotional distress associated with breast cancer diagnosis and treatment. This is consistent with conclusions from a recent meta-analysis, which found that strategies such as avoidance and emotional venting mediate the relationship between illness perceptions and adjustment in illness [56]. Modification of coping strategies may change the relationship between illness perceptions and cancer-related distress, as coping strategies are more amenable to change than illness perceptions [57].

The importance of support from family members in enhancing coping mechanisms further underscores the role of social support in stress management among breast cancer survivors, aligning with SCT’s emphasis on the importance of social support in coping with stressors. Our results are consistent with the hypothesis that people with high levels of social support have better health outcomes than those with low social support, irrespective of stress [58].

The narratives highlight participants’ engagement in health-promoting behaviors such as regular self-examinations and seeking medical advice for symptoms, underscoring their proactive approach to managing their health. For example, one participant’s experience of discovering a lump through self-examination demonstrates the importance of early detection in managing breast cancer effectively.

The psychological and emotional well-being of participants was influenced by factors such as perceived susceptibility, perceived benefits of treatment, and support from family and healthcare providers. Participants expressed feelings of empowerment and resilience following their diagnosis, emphasizing the positive impact of effective medical interventions and emotional support on their psychological well-being. While the narratives primarily focus on the psychosocial aspects of breast cancer survivorship, participants’ accounts indirectly reflect the importance of timely diagnosis and treatment in improving physical health outcomes. Early detection through self-exams and prompt medical consultation contributed to better treatment outcomes and potentially improved physical health among survivors.

### Implications for intervention advancement

This section outlines practical and culturally tailored interventions based on our study’s findings, offering specific, actionable steps to support Iranian breast cancer survivors. To enhance early detection, we recommend developing community-based education programs that focus on regular self-examinations and screenings. Collaboration with local health centers, religious institutions, and women’s groups is crucial for effective dissemination. Educational materials should be designed to resonate with cultural values, making them both accessible and relevant to Iranian women.

Strengthening psychosocial support is another critical area. We propose establishing culturally sensitive support groups for Iranian breast cancer survivors, facilitated by professionals trained in culturally appropriate care. These groups should provide emotional support, share coping strategies, and address the challenges of living with breast cancer. Involving family members in these groups can further enhance support at home, reflecting the cultural importance of family in patient care. Addressing financial and logistical barriers is also vital; implementing government-funded or NGO-supported programs to subsidize treatment costs and establishing mobile clinics in rural areas can alleviate financial burdens and improve access to necessary healthcare services.

We believe that nurses can play a crucial role in advocating for these systemic changes. Specifically, nursing interventions can include educating and supporting patients about available financial assistance programs, coordinating care to ensure that patients can access these resources, and collaborating with other healthcare providers and organizations to advocate for the establishment of mobile clinics in underserved areas.

Incorporating spiritual and religious beliefs into care is essential for providing holistic support. We recommend collaborating with religious leaders and spiritual counselors to integrate spiritual care into treatment plans, training healthcare providers to address spiritual needs, and offering resources on religious practices as coping mechanisms. Additionally, developing cultural sensitivity training programs for healthcare providers and launching media campaigns with culturally appropriate messaging can improve patient-provider communication and breast cancer awareness. Providing culturally tailored cognitive behavior therapy (CBT) and resilience training can further support survivors’ psychological well-being, addressing the unique challenges they face and enhancing their quality of life.

### Limitations and future studies

Despite its contributions, this study has several limitations that warrant consideration. Firstly, the qualitative nature of the inquiry may limit the generalizability of findings to broader populations of Iranian breast cancer survivors. While qualitative research offers rich insights into participants’ lived experiences, it inherently lacks the statistical representativeness of quantitative approaches. Additionally, the sample size and demographic heterogeneity of participants may have influenced the depth and breadth of themes identified, potentially overlooking nuanced differences in experiences among subgroups of survivors. Future studies could address these limitations by employing larger and more diverse samples, encompassing a wider range of sociodemographic characteristics, cancer stages, and treatment modalities, thus allowing for a more comprehensive understanding of the experiences and needs of Iranian breast cancer survivors.

Moving forward, future research endeavors could explore additional factors influencing health behaviors and outcomes among Iranian breast cancer survivors, such as social support networks, access to healthcare services, and cultural beliefs surrounding illness and treatment. Longitudinal studies could offer valuable insights into the trajectory of survivors’ psychosocial well-being and health behaviors over time, enabling researchers to identify key points of intervention to optimize survivorship care. Furthermore, comparative studies examining the effectiveness of various intervention strategies, including educational programs, support groups, and stress management workshops, could inform the development of evidence-based interventions tailored to the unique needs and preferences of Iranian breast cancer survivors, ultimately enhancing their quality of life and long-term health outcomes.

## Conclusion

In conclusion, this qualitative inquiry provides valuable insights into the experiences of Iranian breast cancer survivors and the factors influencing their health outcomes and behaviors. By applying theoretical frameworks such as the HBM and SCT, researchers and practitioners can develop tailored interventions to address psychosocial barriers, enhance coping mechanisms, and improve health outcomes among breast cancer survivors in Iran. Through educational programs, support services, and culturally sensitive healthcare approaches, efforts can be made to empower survivors, promote resilience, and ultimately enhance the quality of life for individuals affected by breast cancer in Iranian communities.

## Electronic supplementary material

Below is the link to the electronic supplementary material.

Supplementary Material 1

## Data Availability

Data is provided within supplementary information files.
