# Supplementary material for: Narratives of resilience: Understanding Iranian breast cancer survivors through health belief model and stress-coping theory for enhanced interventions
Source: BMC Womens Health. 2024 Oct 8;24:552. doi: 10.1186/s12905-024-03383-7 (PMC11459698; doi:10.1186/s12905-024-03383-7)
Supplement: Supplementary file 1 — Supplementary Material 1 [file 12905_2024_3383_MOESM1_ESM.pdf]

S1 File
Interview Protocol

Title: Narratives of Resilience: Understanding Iranian Breast Cancer Survivors through Health Belief Model and Stress-Coping Theory for Enhanced Interventions

Instructions to interviewer

- Greet the participants
- Introduce yourself
- Give background information about the study. Carefully read through the informed consent form and answer any questions. Give information about the interview.
- Assure the participants that he/she does not have to participate if he/she does not want to.
- Ask for approval to participate in the interview.
- Tell the participants that he/she can stop the interview any time they wish.
- Assure confidentiality.
- Make sure the participants know what the tape-recording procedure is. Ask for approval.
- Ask if the participant has any questions before the interview.
- If the participant agrees to take part, please ensure they sign the consent form.
- If the participant agrees to take part, please ensure they sign two copies of the consent form, give them a copy and keep one copy for our records.
- Check the tape-recording equipment.
- Start the interview.

At the start of the interview, do not record respondent's name or any identifier; just indicate the date, time, gender, age and educational level of the person being interviewed. Lastly record where the interview was taking place.

Introduction

- Welcome and thank you for agreeing to participate in this study. Today we are interested in hearing about your experiences as an Iranian breast cancer survivor and how you have coped with and overcome challenges using the Health Belief Model and Stress-Coping Theory.

General Questions

- What motivated you to participate in this study on narratives of resilience among Iranian breast cancer survivors?
- Can you please share a brief overview of your breast cancer diagnosis and treatment journey?
- How did you first learn about your diagnosis and what thoughts and emotions did you experience?
- How did your family and friends react to your diagnosis and how did it impact your coping process?
- How do you perceive your overall health and well-being since being diagnosed with breast cancer?

**Probes:**

Specific Questions

- How do you perceive their risk?
- How serious do you believe the disease to be?
- What positive outcomes do you associate with medical interventions?
- What obstacles do you perceive in seeking treatment?
- What triggers motivate you to seek medical help?
- How confident are you in managing and overcoming breast cancer?
- How do you assess the stress related to their diagnosis and treatment?
- What techniques do you use to handle stress?

**Probes:**

Questions from Health Belief Model viewpoint

- How has your belief in the severity of breast cancer influenced your actions and decisions regarding treatment and self-care?
- Have you ever experienced doubts or uncertainty about the effectiveness of your treatment plan and how did you address them?
- How have your perceptions of the benefits of adhering to medical recommendations affected your recovery process?
- In what ways have perceived barriers, such as financial or social limitations, influenced your ability to follow through with treatment and self-care?

**Probes:**

Questions from Stress-Coping Theory viewpoint

- What coping strategies have you found most effective in managing the emotional and psychological stress of your breast cancer diagnosis?
- Have you experienced any significant sources of stress related to your illness and how have you addressed them?
- How have you found social support from family, friends, and healthcare providers to be helpful in managing stress and maintaining resilience?
- Have you engaged in any mindfulness or relaxation techniques to cope with stress and promote emotional well-being?
- What is the specific support from your close relatives and friends?

**Probes:**

Questions from Enhanced Interventions viewpoint

- Based on your experiences, what do you believe are the most important factors for healthcare providers to consider when supporting breast cancer survivors?

- How can interventions based on the Health Belief Model and Stress-Coping Theory be tailored to better meet the needs of Iranian breast cancer survivors?
- What advice would you give to other breast cancer survivors who are facing challenges and seeking to build resilience in their journey to recovery?

**Probes:**

Closing Question

- Is there anything else you would like to share about your experience?

S2 File

Information Sheet

Title: Narratives of Resilience: Understanding Iranian Breast Cancer Survivors through Health Belief Model and Stress-Coping Theory for Enhanced Interventions

Purpose of the Study

- The purpose of this research study is to explore the experiences and narratives of Iranian breast cancer survivors to understand how they cope with the disease using the Health Belief Model and Stress-Coping Theory. By collecting and analyzing these narratives, we hope to identify ways in which interventions can be enhanced to better support breast cancer survivors in Iran.
- This study aims to develop a nuanced understanding of the coping mechanisms employed by breast cancer survivors.

Study Procedures

- Participants will be asked to share their experiences and thoughts in a one-on-one interview with the researcher.
- The interview will be audio-recorded for accuracy and transcribed for analysis.
- Participants may also be asked to complete a short survey to gather additional information about their health beliefs and coping strategies.

Benefits of Participation

- By participating in this study, you will have the opportunity to share your experiences and insights as a breast cancer survivor, which may help to enhance the support and interventions available to other survivors in Iran. Your participation will also contribute to the advancement of knowledge in the fields of health psychology and oncology.

Confidentiality

- Any information you provide during the study will be kept confidential and will only be accessible to the research team. Your name and any identifying information will be removed from the data to ensure your anonymity.

S3 File
Consent of Informants

Consent Form
I have read and understood the information provided above regarding the research study titled "Narratives of Resilience: Understanding Iranian Breast Cancer Survivors through Health Belief Model and Stress-Coping Theory for Enhanced Interventions." I understand that my participation is voluntary and that I may withdraw from the study at any time without penalty.<br><br>I consent to participate in the study and agree to share my experiences and thoughts as a breast cancer survivor with the researcher. I understand that the information I provide will be kept confidential and used for research purposes only.
Participant Name: ____________________________<br><br>Participant Signature: ________________________<br><br>Date: __________________

S4 File
Transcripts

Participant	Age	Education	Demography	Translated Verbatim
1	32	Bachelor	Has 1 child. Her husband has accompanied her. He is a doctor. Their financial status is good. No family history.	My husband is a medical doctor, and the whole family is familiar with a lot of medical information. In general, my husband tries not to bring work-related matters into family discussions. For the first time, I noticed a change in the shape of one of my breasts. Of course, before that, I had felt pain in the underarm area, but I didn't pay attention to it or take it seriously until we discovered a hard lump in my breast. I always thought mammography was only for women over 40 or those who had reached menopause.<br><br>I was very afraid of having prosthetic surgery. My husband said, "You are important to me, and if you don't want it, that's okay."<br><br>Finally, it was a difficult but necessary decision that I gave in to. Convincing my friends was even more challenging than undergoing the surgery itself.<br>My son didn't want me to go through with the surgery. One day, when I took my 3-year-old child with me, he said, "Mommy, so why just one?"<br><br>I think I was very lucky that my husband is a doctor who understood my situation. If I had discovered this tumor later, the consequences for me and my family would have been much worse.<br><br>Of course, everyone fears such a disease and, like me, can become depressed to the point where they no longer care about anything or anyone and see themselves at the end of the road. But in my opinion, the environment and the people around you can have a significant impact on the stages of diagnosis, treatment, and recovery. At least, this was true for me.

2	43	Diploma	Married with 3 children. Tenant. The husband is a worker. No family history of cancer. Lacks support.	My sister's insistence on screening was a turning point for me. Although I had my own concerns, **it** was her persistence that finally made me go to the doctor.<br><br>A news segment on breast cancer awareness also prompted me to schedule my first mammogram the very next day. I had heard several times that delayed diagnosis is a major factor in the severity of breast cancer and its impact on women's health. The failure to detect breast cancer early significantly increases its severity and worsens the outcomes for women**.** However, I still neglected these warnings.<br><br>After my sister's insistence**,** it was only a few days later that I received the pathology test result, and the world went dark for me. It wasn’t because she forced me to go to the doctor— it was because I had waited too long. I blame myself. I was in a bad situation, and I had lost precious time. Even though the treatment was difficult, my body responded well to the chemotherapy, and I feel good even now, as I talk to you.<br><br>In general, my husband isn’t around much because he's exhausted when he comes home from work, so we don't exchange many words.
3	43	Bachelor in psychology	A single mother with 2 children and, a family history of cancer (father). Her father passed away from cancer, causing concern about mortality. Her brother provided support.	The story of my illness is very complicated. First, I must say that I lost my father to leukemia when I was in high school. That was a very difficult time for me because I was the youngest child, and all my dreams were shattered.<br><br>After I got married and my second child was born, I found out I had breast cancer. I always tell myself, "It's not your fault." I didn’t fully grasp it at that moment, and I cried a lot that night because of my children.

				After that, my husband's behavior changed, and he started teasing me. He rarely came home and spent most of his time at his mother's house.<br><br>My husband believes that cancer is like other contagious diseases, and he doesn't understand that even people who are genetically predisposed to cancer can get it due to a mix of factors beyond anyone's control—it's a matter of luck and many other elements. But my husband wouldn't listen at all.<br><br>This broke my heart, but I was more worried about my children. These marital issues led to constant disputes, and eventually, my husband divorced me, leaving this as my fate.<br><br>Now that my treatment period is over, I am facing financial problems. But my heart is broken, and my doctor says that depression is very bad for my health, warning that this mindset could lead to a recurrence of my illness.<br><br>Unfortunately, psychological services are very expensive, and no one offers me emotional support. My only joy comes from my two children.<br><br>The government has set up an insurance program for female heads of households, but it's not enough, especially combined with my monthly salary. I also need an injection that is very expensive on the black market. In short, I have no choice but to wait and hope.
4	47	Master in Persian language	A single mother with one married child and a grandchild. Divorced before illness, good financial status. Remarried late in treatment.	By chance, we were hanging out with my uncle's aunt, and they suggested that the hospital offers free breast examinations and mammograms. They said, "We're going tomorrow." I said, "I'll come along for fun." We had a lot of fun, and I didn't take it seriously. When I went to get the results, I gave the names of the four people, and the secretary handed me the results and asked,

				"Who is Gulnar?" Then I went to the car where my father and mother were waiting, and they asked, "What did they say?" I said, "Nothing, I didn't see anything." I told my mother that her blood sugar was rising, and she was feeling sick. When my mother got out, I told my father, "Dad, I really do have a mass."<br><br>I had been separated from my husband for a year, and my separation caused my son-in-law to not let my daughter visit me. I cried during all the chemotherapy sessions because I was alone. Everyone in the neighborhood found out about my illness; every day, someone would come and tell me what to eat, what not to eat, or to try something like Lactab. Some even said, "Your husband has cursed you." It drove me crazy; I was getting worse with each passing day.<br><br>At the very beginning of chemotherapy, I took a hairdryer and shaved off all my hair. My stomach and face looked awful. The doctor even told me, "You won't live for another 50 days." That made me deeply sad, but I told myself that I wanted to reunite my family in these last 50 days of my life. I considered these my final days.<br><br>When my daughter told her father about my condition, he responded, "I don't have the energy or money to spend on your mother or her funeral." I was devastated, my mood was terrible**,** and I was crying all the time.<br><br>When I looked in the mirror, I saw my face had changed, and I said to myself, "I don't even have the courage to ask for help anymore." I felt heartbroken and prayed to God, "God, first give me happiness, then heal me."<br><br>I entrusted myself to God. Wherever He takes me, I will follow. Oh, it's such a pity to carry such grief in one's heart.

				I leave everything in the hands of faith.<br>In the path of love, near and far have no meaning.<br>I see you, and I send you a prayer.......<br><br>Suddenly, my mood changed, and I felt hope and joy. It was as if God had spoken to me. I told my mother, "Mom, God told me I will live. Don't worry, I won't die." I decided to go to a poetry gathering during Muharram. At that gathering, there was a man who had proposed to me several times in the past, but I had rejected him. After the poetry reading, he approached me again and said, "I still want to marry you, even under these conditions." I told him, "The doctors say I have only 50 days left to live." Despite that, he stayed by my side.<br><br>During my treatment, I saw a man in a blue coat and blue pants, holding a blue flower and a box of sweets, at the chemotherapy center. At that moment, I remembered a poem by Hafez, and it felt like God was sending me happiness. We had the most beautiful wedding ceremony in the most stunning hall. He asked me to trust him for the remaining days of my life, and to become a vegetarian. I agreed because of the love he showed me. And now, it's been five years since those "50 days," and by God's grace, I'm still alive. I have been able to continue my education and live my best life without any signs or symptoms of my breast tumor. I am living proof of the power of hope.
5	43	Bachelor	Discovered during early marriage while she was pregnant. She works in an office. She is a tenant. Her husband has accompanied her, and the family has provided support.	I never married. My father had tongue cancer, and my mother had a heart problem, and their loneliness made me not think about marriage.<br>I was shot in my hand, and I couldn't do my mother's work. My mom said: "Daughter, we don't have anyone but you. Go to a general doctor."<br>I also went to the insurance. The doctor said**:** "Madam, get an

				ultrasound and bring it to me." When he saw the ultrasound, he said: "Your body is low in vitamins. You will be fine with just one pill."<br><br>I was so happy. I said**,** "Thank God, there is nothing wrong." But the pain lasted more than a month, and it bothered me a lot. I took the ultrasound to the specialist doctor. The doctor said: "Ma'am, you did the ultrasound a month ago, and now you're coming to show me?" I said, "Mr. Doctor, what's wrong?" He said, "Ma'am, go to the hospital quickly. I will write you a letter for admission to see if it can be operated on." I was there when she got breast cancer, and all her hair fell out. She died after the whole cost of treatment, chemotherapy, and radiation therapy.<br><br>When I arrived in the car, my brother said: "What happened, why are you so pale? There must be nothing wrong."<br><br>Chemotherapy made me feel bad. I went to the first session with my brother and my brother's wife. I was in so much pain that the doctor said: "Your wife's anxiety and depression will slow down her treatment," and he told my brother to cut my hair before hair loss became too concerning. To be honest, that night I cried until morning and complained to God a lot. I thought, "I have to take care of two sick people myself. Now, who will take care of me? And who will do the housework if I am not around?"<br><br>My mother had the same thoughts and turmoil, and that caused her to have a stroke during her third chemotherapy session. I had a heart attack, and the world became dark and empty for me. Neither my treatment nor my recovery was important to me anymore. I stayed behind, with my sick father, and I felt utterly alone from everything and everyone.

				At night, when I was feeling down and couldn't cry anymore, I would fall asleep, and someone would inspire me in my sleep: "There is still time to breathe, and life is in someone else's hands."<br><br>During the radiation treatment, my father developed cancer all over his body, and after a while, he died. After my father's death, I became lonely again, and being alone hurts me deeply. I can't sleep in the evenings or at night because of this loneliness, and I'm afraid and worried that if I die alone, who will understand or even notice that I'm gone?
6	42	Bachelor	Has 2 children. Financial status is good. The husband supported her but not much initially. Family history of illness. Housewife.	I always blame myself for my negligence because, in this case, the fear of knowing the truth did not allow me to make the right decision. In addition, some beliefs in Sufi or mystic religion in the society, which are considered values, took away the opportunity to go to the doctor. I always go to doctors for a pap smear at least once a year, but this time I missed it.<br><br>When I experienced severe chest pain, my physician questioned why I waited so long to seek help, pointing out that family history or the severity of pain were not always indicative of breast cancer.<br><br>My pastor's health talk inspired me to prioritize my health. When the physician examined me and diagnosed the disease, I thought, "I want to fight this disease because I've endured something worse than the disease in life."<br><br>Like me, several people mentioned that they had not had any prior tests, and their disease was found unexpectedly. My husband and I decided to undergo the operation so that one day neither my husband nor my children will feel ashamed of me.<br><br>This way of thinking, along with my husband's support, has helped

				me a lot in my treatment and recovery. Basically, the company of relatives is a divine blessing. Thank God, I am much better now, and I have overcome the stage of fear.
7	45	Bachelor	Has 1 child. Discovered during play with the child. Teacher. Financial status is moderate. No family history of illness.	I noticed a change in my breast when I was playing with my 3-year-old son.<br><br>During that time, I self-examined my breast and felt a firm lump that I hadn't noticed before. Despite feeling generally healthy, I decided to see a physician, who recommended surgery to test the lump, though they didn't initially suspect cancer.<br><br>The surgery was not very difficult, but I was always worried about my husband's reaction.<br><br>When my husband came back after the surgery, it was a moment that eased my heart.<br><br>As far as my husband's work is concerned, he helps me both emotionally and at home, like with cooking, but overall, the fact that he has a positive view of my recovery means a lot. You know, he is generally an optimistic person, and that is good.<br><br>Even though I lost my job, he now works two shifts to compensate for our expenses.<br><br>I don't know anyone who has had cancer in our family, and maybe I am the first one. To be honest, I heard a bit of sarcasm from my mother's side of the family, especially my traditional aunts, who think that cancer is a punishment for bad deeds in the world. But I didn't get much support, and I didn't see much open hostility from anyone. I always try to keep my mind busy at home so that I don't think too much.

8	48	Middle school	Has 3 children. Housewife. Tenant. Financial status is poor. Husband and family supported her.	After a close friend's death from breast cancer, I couldn’t ignore the lump in my breast.<br><br>The result of the test was positive, and my doctor said that I came too late because my lymph node was already involved in the cancer. However, I wasn't afraid and said that nothing can get worse than this and that everything has a destiny.<br><br>I accept it... Every time I hear a new article about my illness... I accept it.<br><br>Some individuals, despite feeling pain, tend to consider the illness as fleeting, struggling to accept the reality of their condition. I came to the conclusion that even though I visited the doctor late, this is still a new opportunity.<br><br>My husband and my relatives were very effective in giving me morale. In addition, the amazing hope for a good future from my family helped me a lot.<br><br>Needless to say, the nurses and hospital staff were very kind to me. Contrary to the common belief that government hospital staff do not care about patients, I saw and experienced the opposite. Of course, the quality of the rooms and food may be lower than in private hospitals, but honestly, they have compassionate staff.
9	48	High school	Has 2 children. Employed. Financial status is moderate. Owns an independent home.	After my arm went numb, I visited a general practitioner who advised me to get an ultrasound. The result showed no serious issue, and he attributed my symptoms to vitamin deficiency. I felt relieved, but this misdiagnosis delayed the accurate detection of my condition.<br>I found it hard to accept being ill and couldn’t grasp what this illness meant or its consequences.

				My two children did not understand much, but they [saw] me [that I am] sad to some extent.<br>Although my job is financial affairs and I have to be present all the time, I convinced the boss to do my work online so that I don't have financial problems for my medical expenses.
10	37	Bachelor	Has 1 child. Housewife. The husband is an employee. The husband's family did not accompany her. Tenant. No family history of illness.	After my arm went numb, I visited a general practitioner who advised me to get an ultrasound. The result showed no serious issue, and he attributed my symptoms to a vitamin deficiency. I felt relieved, but this misdiagnosis delayed the accurate detection of my condition.<br><br>I found it hard to accept being ill and couldn't grasp what this illness meant or its consequences.<br>My two children did not understand much, but they could see that I was sad to some extent.<br><br>Although my job is in financial affairs and I have to be present all the time, I convinced my boss to let me work online so that I wouldn't have financial problems for my medical expenses.<br>When I realized that I was not feeling well and the doctor ordered the immediate operation, it was then that the whole world changed for me.<br><br>I wasn't thinking about my children or my husband. Instead, I was thinking, "God, what will I do about the prayers and fasts I missed?"<br><br>How can I resolve these religious obligations that are troubling me? My concern was deeply rooted in my religious duties.<br><br>My husband's family is very traditional and religious. When I was admitted to the hospital and later discharged, no one from his family visited me, except our neighbor. I kept asking

				myself, "Islam teaches kindness, so why do people like my husband's family ignore me—not as a bride, but as a human being?"<br><br>My husband understands these issues, but he can't do much beyond offering comfort.
11	27	High school	Discovered while single. Family supported. Engaged in low-income home jobs. Financial status is moderate. Concern about marriage. Removed one breast.	The costs of my surgery and work increased a lot, and because I was single, my father said, "Don't worry, my daughter. Even if my life is over, I will sell everything, but I won't let this damage your beauty." The cost of the breast prosthesis was heavy, but my father sold his car.<br>I was able to get the surgery, but despite all this support, I am afraid that my illness will recur and I will have to put pressure on my father again.<br>The discomfort I felt in my breast became a pressing concern after my aunt's diagnosis.<br>Aside from the fear of survival, my other worry was the appearance of my body, which I have now come to terms with, because I use prosthetics. So, I don't worry much when I am outside the house, which means I no longer feel ashamed.
12	42	Diploma	Has 1 child. Housewife. The husband is an employee. Has own home. No family history of illness.	When I found out... I didn't want to accept being ill at all. I wished that the doctor and his words were just a dream.<br>When I went to the hospital for treatment and **had no one** by my side, I started spinning.<br><br>In the hall of the rooms, I saw the condition of other patients, and that was when I saw women worse off than myself.<br>I kept saying, "Thank God, I can still walk."<br><br>I was very anxious about how my appearance might affect my relationships and social interactions.<br>Support from my family provided significant comfort, making me

				feel that I had made progress in my healing journey. I want to be there for my son as he starts first grade, which **only adds to my stress.**
13	38	Bachelor	Has 1 child. Employee. Illness during COVID-19. Due to disease restrictions, no one accompanied her. Financial status is good. The husband supported her emotionally.	I always took care of myself and followed the health news from TV and magazines and Heli channel on social networks.<br>I used the experience of other people's disease stages and whenever I went to the [medical] doctor's office for treatment.<br>I gained experience from those who have this disease, for example, a woman said during chemotherapy because later<br>Don't feel bad from chemotherapy, take a break and this made me cope better with this disease.<br>My illness coincided with the peak of the Corona pandemic. But it was hard to move. Most of the hospitals are either infected or infected with the virus. In short, I do not wish you this blessing.<br>When a suitor comes to me, we like each other first, and all our standards collide and fall in love.<br>We used to sit together, but when I told them that my breasts had been operated and I had prosthetics, they would go away and never find them again.<br>After every suitor who goes and cannot be found, I used to cry a lot because of my parents"<br>"When I got the result of my sampling and the [medical] doctor told me that your cancer is malignant, I went from the office to home with your child<br>I cried in my arms and when my husband came at night and saw the results of pathology and sonography, he brought me a glass of water and said: … I am glad that you are here now, and God is great, it was there that my heart sank and it was like I was halfway through the treatment.<br>I constantly remind my husband about upcoming installment payments, which is an additional stressor.<br>I always took care of myself and followed health news from TV, magazines, and the Heli channel on social networks.

				I used the experience of other people's disease stages, and whenever I went to the doctor's office for treatment, I gained knowledge from those who had this disease. For example, a woman said during chemotherapy, "Take breaks so you don't feel as bad later," and this advice helped me cope better with the illness. My illness coincided with the peak of the COVID-19 pandemic. It was hard to move around. Most of the hospitals were either infected or dealing with the virus. In short, I wouldn't wish this on anyone.<br><br>When a suitor comes to me, we like each other at first, and all our standards align and we fall in love.<br>We would sit together, but when I told them that I had breast surgery and prosthetics, they would leave and never come back.<br>After every suitor who left and couldn't be found again, I would cry a lot because of my parents.<br>When I got the results of my biopsy and the doctor told me that my cancer was malignant, I went home with my child and cried, holding him in my arms.<br>When my husband came home that night and saw the pathology and sonography results, he brought me a glass of water and said, "I'm just glad you're here now, and God is great." It was at that moment that my heart sank, and I felt like I was halfway through the treatment.<br>I constantly remind my husband about the upcoming installment payments, which adds to my stress.
14	43	High School	Single. No support. Unemployed. Financial status is poor.	The hand pain was bothering me a lot, and I couldn't sleep at night because of it. I went to the health network, and the health doctor said there was nothing wrong with my breasts, but if I was still afraid, I should see a surgeon. That’s when I decided to visit a specialist to check the pain in my hands, neck, and lymph nodes. He said the best thing to do was a mammography and ultrasound. Yes, I scheduled an appointment, though with a slight delay, and went two days later. I went alone to get the results while my husband and my 5-year-old

				daughter waited in the car. When the lab doctor came to give me the results, he said, "Ma'am, you need to see a specialist immediately. You have an issue with your chest." My whole body went numb, my blood pressure dropped, and I collapsed on the ground. My husband, seeing me take a long time, eventually entered the lab. The doctor told him the news wasn't good. My condition was terrible, and I got into the car, tears streaming down my face as we drove. My body felt frozen, and I couldn't speak. My child kept saying, "Mom, didn't you say you'd take me to buy some toys?"<br><br>When my husband's family found out about my illness, it was as if I had a contagious disease, and none of them spoke a word to me. When they called my mother-in-law and father-in-law, they said, "Look what trouble you've brought upon our son! There's no point in spending money on this." They claimed that no one in their family had ever had cancer**,** and even the children at school told my child, "Don't come near us, our mother said you might get us sick from your mom." From that moment, my husband's behavior changed significantly. We started having a lot of problems. I was already battling my illness, and my daughter was getting depressed because of the stress at home. My husband didn't have the energy **to** pursue his divorce, and I had to go up and down the court stairs for chemotherapy. Meanwhile, my little one was in elementary school, and I had to help him, while all the housework fell on me, with no one to help or take care of me.<br><br>I had 10 sessions of chemotherapy. I had no one with me—no daughter, no family, no husband. The affection my husband once showed me in the hospital vanished. I went to treatments with significant delays, feeling worse and worse. During the chemotherapy, my whole body and back hurt, and I couldn't sit or walk. Every time I rested my head on the pillow, fistfuls of my hair would fall onto the blanket. I wore a scarf to cover my head so no

				one would see my hair falling out. Only my little child cared, asking me, "Mom, why did you become so ugly? You don't have eyebrows anymore. I don't like you, and the other kids don't like me because of you." Those words hurt me deeply. Instead of receiving sympathy from my relatives, I was met with insults. They would ask, "What did you do to deserve this? Your son-in-law was taken from you."<br><br>During chemotherapy, the skin on my face began to peel, and I looked terrible. I was also taking many medications. After kissing my child, his face turned red, and he had what looked like an allergic reaction. I took him to a specialist, who said that the side effects of chemotherapy had affected my skin, and I needed to be cautious. Bad things kept happening to me from every direction, and I completely lost my spiritual connection with God. Although I didn't speak about my condition much, I was determined to get better.<br><br>After chemotherapy, I had my breast removed, and the stitches burned intensely. I was in so much pain from the disease. My sister-in-law had gone for tests, and the doctor diagnosed her with colon cancer. I was already overwhelmed, and then they started saying, "His daughter-in-law's illness is because of her mother-in-law—it's all her fault." I was already suffering, and now I was blamed for my sister-in-law's illness. My mother-in-law and father-in-law constantly taunted me, and my mood worsened.<br><br>Ever since chemotherapy, and especially after my appearance changed, my husband has lost all patience. He works for no real reason and stays out most of the time. We no longer sleep next to each other, and when it comes to intimacy, it feels like he doesn't care anymore. I feel like he no longer loves me, and I'm ashamed in front of him.

15	29	Associate Degree	Engaged during illness. Aunt had a previous illness and, a family history. Husband supported. Did not inform families during treatment. No children yet.	When I was engaged, and even though I was young—I was almost 20 years old—I found out about my illness. Fortunately, I have an understanding husband, and he did not want anyone from his family to know.<br><br>I was not ready to learn about the treatment experiences of others because it was painful for me to confront my illness.<br><br>Here’s how I found out that I have breast cancer: My condition was bad; I tried to know less about my illness and told myself, “It's just a cold; I'll get better soon.”<br><br>Every day, I became more and more unwell, and sometimes my bones ached. In short, I was thinking about everything except cancer, especially as a young person getting married.<br><br>When my doctor asked me to do a general checkup, the first series of tests did not show anything special. My doctor said that probably the laboratory I had gone to was not of good quality and referred me to another testing center, which finally gave a positive result.<br><br>My fiancé was with me at every stage, and after that hard day, he gave me strength and told me that he would help me in every way to regain my health. You know that it is very difficult to hide this, especially from your husband’s family, who are curious about you. But anyway, those difficult times are over, and we still have each other, or we will call on each other now.
16	27	Bachelor	Aunt was ill. Discovered illness during the engagement. Financial status is moderate. Husband supported. Has income. Her husband is a teacher.	I was married when I noticed that my breast was not its normal shape, and it was painful. I always thought that breast cancer doesn't hurt, and everyone says it's silent. People often say women don’t get it until they’re married or have children.

				Once, when my chest was painful and swollen, I went to the doctor. She examined me and scolded me, saying, "Girl, why did you wait so long? You have a family history, don't you?"<br><br>That was a year ago, and the doctor told me that pain or no pain, age doesn't matter. You have a genetic risk, and you need to act quickly. The doctor called my husband and said, "You need to act fast; otherwise, it will spread to other parts of her body. We are very worried."<br><br>I'll never forget what it was like having to tell our families, especially my husband's family. We didn't want them to know. After we came back to my husband's house, he told his parents, "I have a business trip, and I'll be gone for two weeks. I want to take Miriam [our daughter] with me so I won't be alone."<br><br>Saying that gave me a lot of comfort. It's been two years since the diagnosis, and thank God I have recovered.<br><br>But now I worry about having a baby.<br><br>When I was diagnosed, I resolved to fight the disease, drawing strength from overcoming previous life challenges.
17	35	Diploma	Single [divorced]. Has two children in the family. Financial status is good. Family supported. Unemployed.	During my difficult period of chemotherapy**,** there wasn't a moment when I didn't thank God. I said, "God, why are you doing this to me? I was always mindful of what is halal and haram." But once, when I went to the hospital for treatment, I saw a woman with two babies in her arms. She couldn't walk, and there was no one to take care of her babies.<br><br>I said to myself, thank God, the people around me, especially my husband, support me and don't let me suffer. My family never

				reacted badly to me and gave me a lot of comfort. But I realized that they prayed for me a lot during their prayers and supplications.<br><br>Once, my elder brother said, "Sister, don't worry at all. God is with you, and you will get better soon. Even if it's necessary, I will go to the desert for forty nights and seek refuge, asking God to give you intercession."
